# Music and Lectures

**Published:** 1889-11

**Authors:** 


### MUSIC AND LECTURES.

On Wednesday evening, November 20th, is to be given a free lecture by Dr. Babbitt, at the concert room of Prof. J. Jay Watson, 15 East 14th Street, illustrating his great discoveries with reference to sunlight and other of the grand healing agencies of nature. Prof. Watson will furnish his delightful music for the occasion.

Dr. Babbitt is dean of the New York College of Magnetics, and this lecture is designed to be iniatory to a course of evening lectures which, with the aid of some private instruction, will enable those who attend the course to take a diploma of the college. Dr. Babbitt can be seen at his office, 50 Union Square, New York.

